# Surface mechanical properties as a potential upstream contributor to exercise-associated muscle cramp susceptibility: a hypothesis linking athlete–surface interaction, neuromuscular fatigue, and altered neuromuscular control

**DOI:** 10.3389/fspor.2026.1848906

**Published:** 2026-08-07

**Authors:** Michael Hales, John David Johnson

**Affiliations:** 1 Department of Health Promotion & Physical Education, Kennesaw State University Kennesaw, Kennesaw, GA, United States; 2 Department of Exercise Science & Sport Management, Kennesaw State University Kennesaw, Kennesaw, GA, United States

**Keywords:** altered neuromuscular control, cramp threshold, electromyography, Golgi tendon organ, muscle spindle, playing surface

## Abstract

Exercise-associated muscle cramps (EAMC) remain a common, disruptive, and incompletely explained phenomenon in sport. Traditional explanations have emphasized dehydration and electrolyte imbalance; however, accumulating evidence indicates that these factors alone do not adequately explain many cramping events. The altered neuromuscular control theory provides a more plausible physiological mechanism, proposing that fatigue disrupts the balance between excitatory input from muscle spindles and inhibitory feedback from Golgi tendon organs. While this framework explains the immediate pathway leading to cramp, it does not sufficiently identify the upstream factors that initiate the fatigue state. We propose that the interaction between the athlete and the mechanical properties of the playing surface represents an underrecognized upstream contributor to EAMC. Exposure to unfamiliar or mismatched surface mechanical properties is proposed to alter muscle recruitment patterns, increase localized neuromuscular demand, and accelerate fatigue development. This fatigue-related state may increase susceptibility to the excitatory–inhibitory imbalance associated with EAMC in athletes operating near an individual cramp threshold. Evidence from studies examining surface stiffness, energy dissipation, energy return, and myoelectric activity support the premise that surface characteristics influence neuromuscular behavior, metabolic demand, and performance output. When athletes train and compete on surfaces with differing mechanical properties, the resulting mismatch in neuromuscular preparation may increase susceptibility to EAMC. Accordingly, aligning training conditions with competition surfaces, or optimizing footwear–surface interaction, represents a plausible risk-reduction strategy that requires further experimental validation. This hypothesis integrates biomechanical and neuromuscular perspectives and provides a testable framework for future experimental research aimed at identifying modifiable risk factors for EAMC.

## Introduction

Exercise-associated muscle cramps (EAMCs) remain a persistent, disruptive, and incompletely explained problem in sport. Consider a competitive event in which an athlete is forced to abruptly exit play at a critical moment due to a painful, involuntary muscle contraction. This scenario is familiar to coaches, athletic trainers, and athletes across many sports, yet the underlying cause of EAMC remains debated and unresolved. The debilitating pain and spasmodic muscle contraction associated with EAMC can impair performance immediately, with recovery time that is often unpredictable and highly variable (1–5).

For decades, dehydration, electrolyte imbalance, and environmental conditions have been cited as primary explanations for EAMC (6). This paper does not dismiss the importance of hydration practices before, during, and after exercise, and proper hydration guidelines should continue to be followed, particularly in challenging environmental conditions. However, the longstanding view that dehydration is the primary cause of EAMC has been increasingly questioned, and recent evidence suggests that hydration and electrolyte explanations do not adequately account for many cramping events (5, 7–9). The continued emphasis on hydration and electrolyte imbalance as primary causes of EAMC may overlook critical neuromuscular and biomechanical factors that better explain many cramping events observed in sport.

A more plausible explanation has emerged in the form of altered neuromuscular control (10, 11). This theory developed from laboratory-based work using surface electromyography (sEMG) and related neuromuscular investigations examining spinal reflex activity during fatigue and cramping (10, 11). These studies suggest that cramping is rooted less in whole-body fluid loss and more in local neuromuscular dysfunction associated with fatigue (12), heat (13), or decreased energy supply (14). In other words, the immediate physiological pathway to EAMC is better explained by a fatigue-related disturbance in neuromuscular regulation than by dehydration alone.

The present paper extends this framework by identifying a potential upstream factor contributing to neuromuscular fatigue. If altered neuromuscular control explains how a cramp occurs, then a more important question remains: what initiates the neuromuscular disturbance in the first place? We propose that the interaction between the athlete and the mechanical properties (MPs) of the playing surface represents an underrecognized contributor to this disturbance. Specifically, exposure to unfamiliar or mismatched surface conditions is proposed to influence muscle activation and recruitment patterns, accelerate localized fatigue, and place the neuromuscular system in a state that favors cramp development. The etiology, therefore, involves more than dehydration, electrolyte loss, or generalized fatigue; it also involves the way in which unfamiliar surface demands alter the athlete's movement strategy and neuromuscular loading during sport activity (15–17).

This hypothesis is grounded in prior work showing that surface MPs can influence physiology, biomechanics, and myoelectric activity during sport-specific tasks (16, 17). Proper neuromuscular preparation may therefore require more than conditioning in the general sense; it may require preparing on surfaces with similar mechanical characteristics to those encountered in competition. The interaction between the athlete and the playing surface introduces an environmental variable with the potential to influence muscle physiology, fatigue development, and ultimately EAMC risk. While the present framework is most directly applicable to running and field-based sports, the underlying principles may extend to other activities involving repeated high-intensity locomotion under variable surface conditions. To understand this rationale, the physiological pathway leading to cramp must first be reviewed.

The proposed framework describes a sequential mechanistic pathway rather than an established causal relationship. Specifically, unfamiliar surface mechanical properties may alter muscle recruitment strategies, increasing localized neuromuscular demand and accelerating fatigue development. Fatigue-related changes in sensory feedback from muscle spindles and Golgi tendon organs may then contribute to altered neuromuscular control and increased susceptibility to EAMC. Although each component of this pathway is supported to varying degrees by existing literature, the direct relationship between surface mechanical mismatch and EAMC occurrence remains untested and represents the primary research question generated by this hypothesis. This hypothesis further predicts that the effect of surface mismatch is conditional rather than general: it is expected to manifest specifically under conditions of high-intensity, competition-level exertion sufficient to approach an individual's cramp threshold, and following repeated or sustained exposure rather than a single novel bout. A brief, low-intensity, voluntary exposure to an unfamiliar surface, such as recreational activity on sand, would not be expected to produce measurable differences in cramp susceptibility under this framework, since neither the fatigue accumulation nor the loading volume required to disrupt the excitatory–inhibitory balance central to altered neuromuscular control theory would be reached. The absence of existing incidence-level data reflects, in part, the methodological difficulty of isolating surface mechanical properties as a variable in field-based cramp surveillance, where hydration, heat, training load, and individual cramp history are typically confounded.

## Hypothesis

We hypothesize that exposure to unfamiliar or mismatched surface mechanical properties alters muscle recruitment patterns and increases localized neuromuscular demand, which may accelerate fatigue development and increase susceptibility to exercise-associated muscle cramps. More specifically, when athletes train under one set of mechanical conditions but compete under another, the neuromuscular system is exposed to unaccustomed loading patterns, altered stiffness demands, and different energy-loss characteristics. These changes may contribute to premature local fatigue, disrupt the balance between excitatory and inhibitory neuromuscular input, and increase neuromuscular excitability associated with EAMC susceptibility. Therefore, surface mechanical properties are proposed to function as a plausible upstream contributor to the fatigue-related neuromuscular state associated with EAMC susceptibility. This hypothesis is testable through controlled comparisons of neuromuscular activity, fatigue development, and cramp incidence under matched and mismatched surface conditions, with surface mechanical properties systematically manipulated to assess their effect on muscle recruitment patterns and fatigue onset.

## Supporting theoretical framework

To support this hypothesis, it is necessary to establish a theoretical framework linking localized neuromuscular fatigue, altered neuromuscular control, and the mechanical properties of the playing surface. The following sections outline the physiological and biomechanical mechanisms through which surface conditions may act as an upstream contributor to exercise-associated muscle cramps.

Accordingly, surface mechanical properties should be considered not as a passive environmental feature, but as an active contributor to neuromuscular fatigue and cramp susceptibility.

### Acute localized muscle fatigue

At the initiation of a task, the involved muscles are strong, coordinated, and capable of producing force efficiently. With repetitive and continuous motion, however, these muscles progressively lose their capacity to generate force and become fatigued over time (18). More precisely, this phenomenon can be described as acute localized muscle fatigue (ALMF), a term used in the present context to denote fatigue that develops within a specific muscle or muscle group during sustained or repeated activity. In this state, the affected musculature gradually loses its ability to function normally, as may occur in the hamstrings or calf musculature (19, 20), ultimately reflecting a condition of localized muscular exhaustion following strenuous or repeated loading.

At the onset of ALMF, neuromuscular control becomes progressively disrupted rather than simply weakened (18). This disruption manifests as altered motor unit recruitment patterns, reduced coordination, and decreased movement efficiency, ultimately impairing the muscle's ability to produce force in a controlled manner (34). In the context of EAMC, this distinction is critical, as fatigue does not merely reduce force capacity, creates a neuromuscular environment that may increase susceptibility to localized cramping under conditions where other risk factors are present.This pattern is well characterized at the level of individual motor units. During repeated submaximal contractions, motor unit recruitment thresholds progressively decline while firing rates increase, reflecting a compensatory increase in central excitatory drive as previously recruited units fatigue and additional units are recruited to sustain force output (22, 23). Although this body of work has not examined playing-surface mechanical properties directly, it establishes the physiological mechanism by which cumulative submaximal loading, of the kind imposed by repeated locomotion on a given surface, could progressively shift motor unit recruitment strategy well before overt fatigue or measurable force loss becomes apparent.

**Figure 1:**
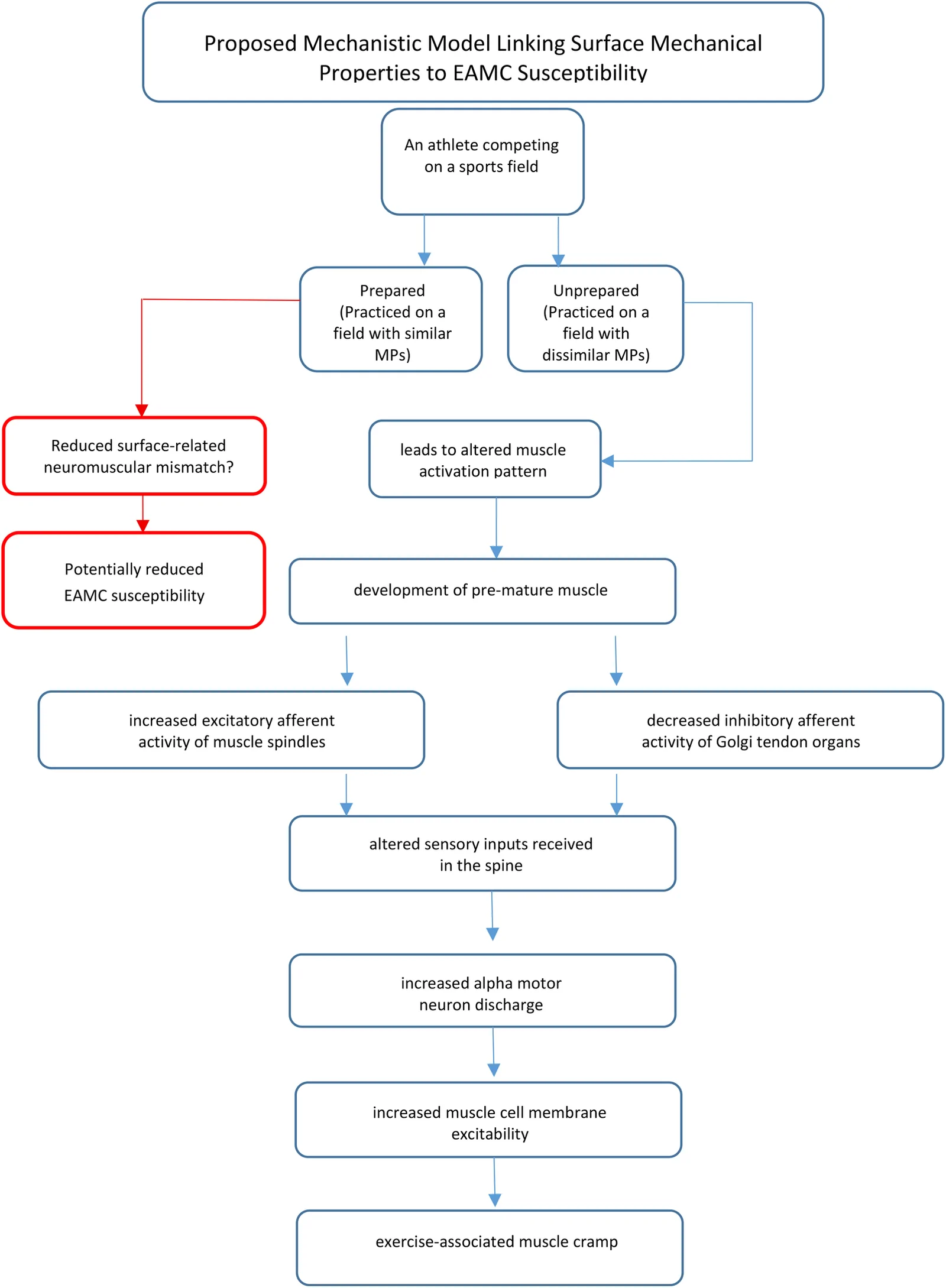
Flow chart depicts an unexpected source (playing and practicing on surfaces with different mechanical properties), the physiological dysfunction (altered neuromuscular control), and ultimate result (exercise-associated muscle cramp). Conversely, competing and preparing on a field with similar mechanical properties could produce a non-EAMC condition.

Two precursor scenarios are especially relevant. The first is the more obvious case: an abrupt increase in the volume or intensity of high-effort activity. The second, and more novel, involves performing a familiar volume of high-intensity activity under unfamiliar surface conditions that impose different mechanical demands. In this latter case, the athlete is physically conditioned for the activity itself yet insufficiently prepared for the specific mechanical demands imposed by the competition surface (Figure 1). This distinction is important, because it suggests that fatigue risk is not governed solely by workload, but also by the mechanical context in which that workload occurs.

Support for this concept is provided by studies examining muscle activation patterns across different running surfaces (24–26). More specifically, one research study reported that myoelectric activity during sprinting differed when athletes performed on sport fields with different MPs under both pre-fatigue and post-fatigue conditions, with differences of 11% for males and 9% for females (17). Their findings demonstrate that the MPs of a playing surface influence muscle recruitment patterns by as much as 50% and influence the onset of muscle fatigue by approximately 30% (17). Although these findings are supported by a limited number of controlled studies, they are consistent with a broader body of literature demonstrating that surface-dependent loading influences neuromuscular and metabolic responses (27–29). If fatigue is accelerated by the MPs of the surface, then addressing the player–surface interaction becomes a direct and mechanistically justified means of reducing premature local fatigue.

This concept is further reinforced by independent work demonstrating that surface stiffness influences not only muscle activation magnitude but also the broader neuromuscular strategy used to regulate lower-limb stiffness. Using a controlled hopping paradigm, surface compliance was shown to shape both preferred movement frequency and the underlying electromyographic behavior of the foot and ankle musculature, with limb-stiffness regulation adjusting systematically as surface stiffness changed (30). Although this work was conducted using a repeated bouncing task rather than field-based sport movement, it provides independent confirmation, beyond the authors' own prior work, that the neuromuscular system actively reorganizes its recruitment and stiffness-regulation strategy in response to surface mechanical properties.

Sequential contractions of an isolated muscle can produce fatigue because of inadequate conditioning, abrupt increases in stimulus volume, or extended duration of the activity (31). During neuromuscular fatigue, the muscle spindles and Golgi tendon organs (GTOs) become especially important because these sensory structures regulate excitatory and inhibitory feedback (12). Fatigue increases excitatory afferent input from the muscle spindles and decreases inhibitory afferent activity from the GTOs. The coupling of these events leads to altered neuromuscular control at the level of the spinal cord (32, 33). These changes disrupt normal neuromuscular regulation and place the system in a state that favors cramp development. Therefore, any factor that accelerates localized neuromuscular fatigue, regardless of its origin, increases the likelihood of disrupting the excitatory–inhibitory balance that precedes cramp development.

### Alpha-motor neuron and GTO coupling

According to altered neuromuscular control theory, an acute localized EAMC occurs when the alpha motor neuron becomes excessively excited (32, 34). Alpha motor neurons represent the final common pathway for activating skeletal muscle fibers, and their output must be balanced by inhibitory regulation for normal contraction and relaxation to occur. This regulation depends on the coordinated interaction between the muscle spindle and the Golgi tendon organ (GTO) (12, 35).

The muscle spindle functions as a length-sensitive receptor that promotes muscle contraction, whereas the GTO provides inhibitory feedback that regulates muscular tension and facilitates relaxation (12, 35). Normal movement depends on the coordinated operation of both systems. In simple terms, the muscle spindle promotes continued contraction, while the GTO limits excessive contraction and permits relaxation. When both systems function appropriately, movement remains controlled and efficient.

During fatigue, however, this balance becomes disturbed. Muscle spindle activity increases as the neuromuscular system attempts to maintain force production, while inhibitory input from the GTO declines (12, 35). The result is an overexcited alpha motor neuron, persistent contraction of the fatigued muscle, and ultimately the development of a cramp. This mechanism is central to the altered neuromuscular control theory and provides a compelling physiological basis for EAMC that is more consistent with many field observations than the traditional dehydration model alone. Importantly, this mechanism is initiated and amplified by localized neuromuscular fatigue, reinforcing the role of fatigue as the critical upstream condition in the development of EAMC.

Independent physiological evidence lends additional support to the plausibility of this pathway at the level of the sensory receptor itself. Work examining the muscle spindle directly has shown that its afferent firing is sensitive to the mechanical compliance of the tissue in series with it; increasing series compliance has been shown to attenuate muscle spindle firing rates during stretch, reducing both the dynamic and static components of the response (36). This finding is notable because it demonstrates that mechanical compliance can modulate excitatory afferent drive independent of fatigue-related metabolic changes, rather than through fatigue alone. This relationship, however, does not appear unconditional. Work examining the triceps surae found that differences in musculotendinous stiffness did not meaningfully alter spinal stretch reflex sensitivity once adequate muscle pretension had been established, suggesting that the influence of compliance on spindle behavior may depend on the prevailing loading or pretension state of the muscle at the time of stretch (37). Applied to the present hypothesis, these findings do not demonstrate that playing-surface compliance directly alters spindle behavior during sport; rather, they establish that mechanical compliance is a physiologically credible modulator of the excitatory afferent pathway central to altered neuromuscular control theory, independent of, and potentially additive to, the fatigue-mediated pathway already proposed.

### Altered neuromuscular control theory

EAMCs are not classified as a primary muscle disorder, but rather as a dysfunction of neuromuscular regulation characterized by an imbalance between excitatory and inhibitory sensory input. Neurons propagate electrical signals in the form of action potentials, forming the communication network that coordinates skeletal muscle function (38). Within this framework, altered neuromuscular control has been described by two primary viewpoints: a peripheral origin theory and a central, or spinal, origin theory.

The peripheral origin theory proposes that abnormal excitation at the terminal branches of the motor nerves leads directly to cramping in the muscle itself (39). The central, or spinal, origin theory proposes that involuntary muscle contraction results from altered neural signaling to and within the spinal cord as a consequence of muscle fatigue (11, 39). While these theories differ in emphasis, both place neuromuscular signaling at the center of cramp development.

Within the altered neuromuscular control framework, EAMC occurs when increased alpha motor neuron discharge develops due to an imbalance between excitatory input from muscle spindles and reduced inhibitory feedback, particularly from the GTO, which normally dampens excessive contractile excitability (40). When GTO-mediated inhibition is suppressed, an imbalance develops between excitatory input from muscle spindles and inhibitory spinal input reaching the muscle. The consequence is increased muscle cell excitability and, ultimately, cramp development (11). Collectively, this body of work demonstrates that fatigue-related altered neuromuscular control provides a more comprehensive explanation for the cause, treatment, and prevention of many EAMCs than dehydration-focused explanations alone (41).

The remaining question is not whether altered neuromuscular control can produce EAMC, Available evidence supports altered neuromuscular control as a leading explanatory framework for many EAMC events. We propose that one such condition is the athlete's interaction with a playing surface whose mechanical characteristics differ from those to which the athlete is accustomed, thereby imposing unaccustomed neuromuscular demands. Under these conditions, altered muscle recruitment patterns and accelerated fatigue development highlight how surface-dependent loading conditions may accelerate the development of the neuromuscular imbalance associated with EAMC development.

### Surface mechanical properties within a multifactorial model of exercise-associated muscle cramps

Exercise-associated muscle cramps are unlikely to result from a single isolated mechanism. Current evidence suggests that EAMC develops through the interaction of multiple intrinsic and extrinsic factors, including previous history of cramping, training status, exercise intensity, fatigue accumulation, environmental conditions, pacing strategy, muscle damage, hydration status, electrolyte balance, and individual susceptibility.

Several of these factors are supported by direct epidemiological evidence independent of the present hypothesis. In a case-control study of 433 Ironman triathletes, a self-reported history of EAMC was independently associated with faster relative race intensity and a positive family history of cramping (42), and a related genetic case-control study reported that variation in the COL5A1 collagen gene was associated with EAMC history among endurance athletes (43), suggesting a heritable component to individual susceptibility. In a field-sport-specific cohort, a prospective study of rugby league players identified competition level and prior cramp history, among other factors, as predictors of in-season calf cramping (44). These findings reinforce that surface mechanical properties are best understood as one contributor operating within this multifactorial and individually variable risk profile, rather than as a sufficient cause on their own.

Within this multifactorial framework, surface mechanical properties should not be considered an independent cause of EAMC. Rather, the athlete–surface interaction represents a potential environmental factor that may modify the neuromuscular demands imposed during exercise. An athlete competing on a mechanically unfamiliar surface may experience altered loading patterns, changes in lower-extremity stiffness regulation, and modified muscle recruitment strategies. These adaptations may increase localized neuromuscular demand and accelerate fatigue development, particularly when combined with other established EAMC risk factors.

The proposed model therefore extends existing EAMC theories by identifying surface mechanical properties as a possible upstream contributor that interacts with, rather than replaces, established physiological and environmental influences.

### Muscles most susceptible to EAMCs

Research examining running and field-sport movement has identified differences in biomechanical behavior at the ankle, knee, and hip when athletes perform on surfaces with different mechanical properties (45–49), particularly when those surfaces are unfamiliar to the musculoskeletal system (27, 29, 50–52). These findings indicate that muscles demonstrating abnormal stiffness responses relative to the individual are especially vulnerable to EAMC.

This is particularly relevant for bi-articular muscles such as the hamstrings, quadriceps, and calves, which span two joints and play a central role in force transmission, stabilization, and rapid movement (46, 47). Surface MPs, including impact absorption and related stiffness properties, directly influence lower-extremity range of motion and joint behavior (45–47, 49). In general, a stiffer surface reduces ankle range of motion, whereas a softer surface may promote greater motion at the hip and knee during running and cutting movements (29, 52). Such changes alter local muscle mechanics and contribute to the abnormal tension conditions associated with cramp susceptibility.

Although EAMCs are not limited to bi-articular muscles, these muscles are among the most common sites of cramping in athletes competing in field- and court-based sports (39). If such athletes routinely practice and compete on surfaces with unfamiliar MPs, their lower-extremity musculature is repeatedly exposed to altered tension patterns and recruitment strategies. For example, when a muscle contracts in a shortened position or through a reduced arc of motion, GTO-mediated inhibition is diminished due to altered muscle tension (7). If injury, restriction, or fatigue further reduces range of motion, the resulting neuromuscular imbalance becomes more pronounced.

This same physiology explains why stretching can relieve an active cramp. Lengthening the affected muscle, either passively or by moving it through its full range of motion, alters muscle tension and increases inhibitory input from the GTO to the alpha motor neuron, thereby promoting relaxation (7, 39, 53). This point is important because it links the immediate treatment response directly to the neuromuscular mechanism underlying the cramp. It also suggests that preventing premature EAMC requires attention not only to the muscle itself, but also to the mechanical environment influencing that muscle's behavior. Because surface mechanical properties directly influence joint behavior, muscle stiffness, and recruitment patterns, they represent a plausible contributor to the localized fatigue and neuromuscular imbalance observed in EAMC, particularly within muscles exposed to repeated or unaccustomed loading patterns.

### Abnormal muscle recruitment patterns

A critical extension of the altered neuromuscular control theory is the identification of sport-specific conditions that alter muscle recruitment patterns. Among these, the mechanical properties of the playing surface represent a consistent and often overlooked variable. Historically, many sport professionals have attributed EAMCs primarily to physiological responses associated with heat, hydration status, or electrolyte loss (7, 39). A newer proposal is that the MPs of a playing surface alter muscle activation patterns in a manner that promotes premature neuromuscular fatigue and contributes to the initiation of EAMC (16, 29). Several studies examining the player–surface interaction provide direct support for this concept (16, 29, 54–58).

Importantly, exposure to mismatched surface conditions is not a rare occurrence in sport. Athletes frequently transition between practice and competition environments that differ in surface stiffness, energy restitution, and overall mechanical behavior. These transitions often occur without sufficient neuromuscular adaptation, effectively exposing the athlete to unfamiliar loading conditions during high-intensity activity. Under these circumstances, the neuromuscular system is required to rapidly adjust recruitment strategies, increasing the likelihood of premature localized fatigue.

One study analyzed muscle activation patterns using sEMG during sprinting and demonstrated that the MPs of the sport field influence myoelectric recruitment patterns (16). The testing fields were quantified using ASTM protocols measuring force reduction, vertical deformation, and energy restitution. Athletes completed sprint trials under pre-fatigue conditions and again following a high-intensity sport-specific fatigue protocol consisting of running and agility drills (16). Course completion times differed by 5.2% between field types, while footwear was controlled by providing all participants with the same model of shoe (16). These design features are important because they strengthen the argument that field MPs themselves, rather than uncontrolled footwear differences, contributed to the observed effects.

The practical findings from this line of work are especially noteworthy. First, the MPs of the field influenced muscle activation patterns even before fatigue was induced. Second, field MPs combined with neuromuscular fatigue produced sEMG patterns that differed from those observed under pre-fatigue conditions, with post-fatigue differences reported at 13%. Third, biceps femoris activity differed substantially between males and females, by approximately 50%, indicating that the physiological response to surface conditions is not uniform across groups (17). The stiffer field demonstrated the least amount of energy loss and also produced the fastest sprint times. Together, these findings support the proposition that neuromuscular activation patterns are influenced by surface MPs and that these influences matter not only for performance, but also for fatigue development and EAMC susceptibility. In this context, ‘energy loss’ denotes the portion of mechanical energy that is not returned during athlete–surface interaction and is instead dissipated through viscoelastic deformation and other non-recoverable pathways (e.g., heat and sound). This convention is consistent with terminology used in sport surface mechanics and does not imply a violation of energy conservation.

This issue becomes especially relevant in real-world sport settings. In many programs, athletes do not practice and compete on the same field. They may practice on natural grass and compete on synthetic turf, or vice versa. In some cases, practice and competition fields may be made of the same general material yet differ substantially in quality, wear, compaction, and therefore MPs. Budget constraints and facility limitations often make these mismatches unavoidable. Research has shown that fields composed of the same broad material category can differ substantially in both surface quality and MPs (54–58). This distinction is critical because field composition alone is not sufficient to characterize the athlete's mechanical environment.

This point bears directly on how the present body of evidence should be weighted. A limited number of investigations examining surface effects on muscle activation, fatigue, or performance have independently measured the mechanical properties of the surfaces tested, using standardized protocols such as ASTM force reduction, vertical deformation, and energy restitution testing (16, 17, 54–57). The majority instead classify surfaces by type or material category (e.g., artificial turf vs. natural grass) without confirming the underlying mechanical properties of the specific fields used, an approach that treats surface type as a proxy for the mechanical variables of actual interest. Because fields of the same nominal type can differ substantially in measured properties, and fields of different types can sometimes exhibit similar properties, this proxy is imprecise and may partly account for inconsistent findings across the surface-performance literature. The present hypothesis draws most heavily on investigations, including the authors' own prior work, that directly quantified surface MPs alongside neuromuscular or performance outcomes, reflecting a deliberate preference for studies meeting this methodological standard rather than a lack of independent corroboration in the broader literature.

Collectively, these findings demonstrate that surface mechanical properties influence muscle activation patterns, fatigue development, metabolic demand, and performance outcomes (16, 17, 27, 28). When interpreted within the framework of altered neuromuscular control, these effects collectively demonstrate how surface mechanical properties systematically influence neuromuscular demand and fatigue development, establishing a mechanistic link to EAMC.

### Mechanical properties, energy loss, and performance demand

Building on these findings, data-driven evidence demonstrates that time-dependent surface properties elicit specific myoelectric activation patterns (16, 17). The most relevant of these properties include force reduction, vertical deformation, and energy restitution. Energy restitution refers to the amount of energy returned to the athlete from the playing surface. However, energy return should not be viewed as a universal performance benefit. For returned energy to enhance performance, it must fall within the body's capacity to receive, manage, and convert that energy into useful mechanical output (59, 60).

For this reason, the phrase ‘energy return’ is at times less useful than the concept of reduced energy loss. A surface that minimizes excessive energy dissipation allows more efficient movement, but only if the athlete is prepared for the accompanying loading characteristics. Surface mechanical properties influence how mechanical energy is stored, dissipated, and returned during athlete–surface interaction. However, the physiological consequence of these properties depends on whether the athlete is adapted to the imposed loading environment. Therefore, surface characteristics should not be interpreted as universally beneficial or detrimental but rather as mechanical conditions that interact with athlete-specific neuromuscular capacity and preparation.Applied to performance, these relationships demonstrate that a sport surface with specific MPs imparts loading characteristics that are either compatible or incompatible with the viscoelastic behavior of the human body (28). Studies examining surface stiffness, metabolic energy expenditure, and mechanical energy loss report meaningful differences across surfaces (16, 27, 28). Surfaces associated with less energy loss are generally linked to lower energy expenditure and improved performance efficiency. However, this does not mean that the ‘fastest’ or ’stiffest’ surface is universally optimal. Performance benefits depend on the athlete's ability to adapt to the loading characteristics of the surface. When this match is absent, the resulting neuromuscular mismatch may increase fatigue and impair performance. Thus, surface mechanical properties influence both performance output and physiological demand.

These studies reinforce the importance of analyzing the athlete–surface interaction rather than treating the surface as a passive background variable. Other investigations examining muscle activity and physiological responses during sprinting and sport-specific activity on surfaces with different MPs report evidence that surface properties influence human performance and neuromuscular behavior (15, 16). From the perspective of EAMC, this is critical because altered muscle recruitment and altered energetic demand directly contribute to premature neuromuscular fatigue.

Another important variable within this system is footwear, as the athlete–footwear–surface interaction ultimately determines how mechanical forces are transmitted, absorbed, and returned during movement. Several studies show that the footwear–surface combination influences outcomes during sprinting, sliding, acceleration, and abrupt change-of-direction tasks (61–64). If field MPs influence physiological response, then footwear amplifies, dampens, or otherwise modifies that influence. For this reason, analysis of EAMC causation in sport must consider the combined system rather than the surface in isolation.

### Common preventative modalities

Established preventative strategies, including appropriate hydration and electrolyte balance, remain essential components of athlete care and should not be disregarded (65–67). These practices play a critical role in maintaining physiological function, particularly in environments characterized by heat stress and high sweat rates. However, evidence suggests that these factors alone do not fully account for many instances of exercise-associated muscle cramps. As such, additional contributors to neuromuscular fatigue and cramp susceptibility should be considered.

EAMCs are common in sport, and a wide range of interventions have been used to treat and prevent them. Treatment and prevention, however, are not equivalent problems. Once an athlete is actively cramping, the event has already occurred, and performance has already been compromised. At that point, immediate treatment is necessary to restore function. Prevention, by contrast, should focus on reducing the likelihood that the neuromuscular conditions leading to cramp developing in the first place.

A number of non-drug therapies have been used in relation to EAMC, including sensory-mediated approaches such as transient receptor potential activation (68–71) and ingestive strategies such as electrolyte supplementation, pickle juice, and mustard (72–74), as well as mechanical interventions including stretching, taping, and other local mechanical interventions (20, 21, 75, 76). Some of these strategies help abort a cramp after it begins, but their preventive value is less clear. Stretching, for example, helps terminate an active cramp by changing muscle tension and increasing inhibitory input, yet prophylactic stretching before competition or at other routine times is not especially effective as a preventive strategy (40). More broadly, current evidence demonstrates that many prophylactic approaches for EAMC are limited at best (77).

If many common prophylactic strategies are ineffective, then what does a more effective prevention model require? Effective prevention requires proper preparation for the competition environment. In practical terms, this involves preparing the neuromuscular system under conditions that closely replicate those encountered during competition, including similar movement patterns, comparable loading rates, and muscle activation patterns reflective of the actual performance setting. This is where the surface hypothesis becomes especially relevant. If competition occurs on a surface with MPs that differ meaningfully from the practice environment, then general conditioning alone is insufficient.

Another relevant concept is cramp threshold. Cramp threshold refers to the amount of electrical stimulation required to trigger a cramp in a given individual (65, 77). This concept aligns with prior work demonstrating that fatigue lowers cramp threshold and increases neuromuscular excitability. This threshold varies between individuals and can be modified over time through appropriate training (40). Individuals with a low threshold may cramp at relatively low workloads, whereas those with a higher threshold tolerate greater neuromuscular stress before cramping occurs. Progressive training under appropriate mechanical conditions may shift this threshold in a favorable direction, much as training influences lactate threshold in anaerobic performance. Within the context of the present hypothesis, exposure to unfamiliar or mismatched surface mechanical properties may accelerate fatigue development and cause an athlete to reach their cramp threshold more rapidly. Additional research is needed, but this concept further supports the role of environment-specific preparation in reducing EAMC risk.

Taken together, these mechanisms emphasize the importance of preparing the neuromuscular system under conditions that closely reflect the mechanical demands of competition.

## Practical application and recommendations

For decades, the sport community has pursued explanations and preventive measures for EAMC. Altered neuromuscular control provides a plausible physiological mechanism, but the practical challenge remains identifying the factors that trigger this mechanism in real sport settings. We propose that playing surface MPs represent one such factor. If the player–surface interaction contributes to EAMC, then the preventive target becomes both more concrete and more mechanistically defensible.

One potential strategy generated by this hypothesis is increased exposure to competition-relevant surface conditions during preparation. When that is not possible, such as when traveling to an opponent's venue, the next practical option involves identifying a footwear–surface combination that optimizes biomechanical and neuromuscular response. Research indicates that both footwear and the playing surface influence myoelectric activity and other physiological outcomes (78). Thus, footwear selection offers a means of partially replicating competition-specific loading and muscle activation patterns when direct exposure to the competition field is not possible.

An analogy helps illustrate this point. If an athlete plans to run a road race on asphalt, it is reasonable to perform at least part of the training on asphalt. Preparing only on a softer rubberized track leaves the neuromuscular system insufficiently prepared for the loading pattern encountered on race day, potentially increasing the risk of premature fatigue or EAMC. Conversely, excessive preparation on a much stiffer surface such as concrete exposes the athlete to loading conditions that are unnecessarily harsh and may increase injury risk. The principle is not that one surface is universally superior, but that preparation must reflect the mechanical demands of competition.

A broader practical application is the establishment of a database of field type and field MPs for local sport venues. Such a database provides schools and teams with useful information when preparing for competition on unfamiliar fields. An initial phase involves testing local high school playing fields using standardized protocols and reporting force reduction, vertical deformation, and energy restitution in a centralized format. Additional phases expand this effort to surrounding counties and eventually the state level. If successful, this provides athletic departments with actionable information that informs training, footwear selection, and preparation strategies in advance of competition.

## Conclusion

Human–surface interaction matters. The present paper proposes that athlete–surface interaction represents one underrecognized environmental factor that may contribute to the fatigue-related neuromuscular state associated with EAMC. The immediate physiological pathway remains consistent with altered neuromuscular control: local fatigue increases excitatory input from muscle spindles and reduces inhibitory input from Golgi tendon organs (11), increases alpha motor neuron excitability (12, 33), and ultimately results in cramp (32, 35). The novel contribution of this paper is the argument that unfamiliar or mismatched surface MPs function as an upstream contributor to that fatigue state by altering muscle recruitment patterns, stiffness behavior, and energetic demand.

This does not repudiate hydration practices or the value of established treatment methods. Rather, it expands the discussion by identifying a sport-specific environmental factor that helps explain why some athletes cramp in situations where dehydration alone is insufficient. The supporting literature demonstrates that surface MPs influence myoelectric activity (16, 17, 27, 28), metabolic response, joint behavior, and performance outcomes (29, 45–47, 49, 52). When these influences are interpreted through the lens of altered neuromuscular control, a plausible and testable pathway can be proposedfrom unfamiliar surface conditions to premature local fatigue and ultimately EAMC.

Accordingly, we propose that practicing and competing on surfaces with similar MPs or using footwear and preparation strategies to better match the competition environment, may reduce one potential source of neuromuscular mismatch associated with EAMC susceptibility. Further research is warranted, but the present theory provides a coherent framework for integrating biomechanics, neuromuscular physiology, and applied sport preparation into a single explanation for an often poorly understood event. This framework challenges traditional assumptions regarding EAMC causation and identifies the mechanical environment of sport as a potentially important contributor requiring further investigation.

### Future experimental validation of the surface hypothesis

Future research should directly examine whether surface mechanical mismatch alters neuromuscular responses associated with EAMC susceptibility. Several experimental approaches could evaluate this hypothesis.

A prospective cohort design could examine whether athletes experience greater EAMC occurrence when competition surfaces differ substantially from habitual training surfaces. Relevant variables would include surface mechanical characteristics, footwear, previous EAMC history, training load, environmental conditions, and competition intensity.

Randomized crossover studies could compare athlete responses to matched and mismatched surface conditions during standardized sport-specific fatigue protocols. Outcomes could include surface mechanical properties, electromyographic activity, muscle activation timing, fatigue-related changes in EMG frequency characteristics, perceived exertion, performance decrement, and recovery response.

Controlled manipulation of surface stiffness, deformation, damping, energy restitution, and traction could determine which mechanical properties produce the greatest neuromuscular response. Cramp threshold frequency testing may provide an important mechanistic bridge by determining whether surface-specific fatigue alters susceptibility to experimentally induced cramping.

Longitudinal studies are also necessary to determine whether repeated exposure and adaptation to unfamiliar surfaces reduces neuromuscular disruption. If athletes adapt to surface-specific demands over time, this would provide additional support for the concept that preparation on competition-relevant surfaces may influence EAMC susceptibility.

## Data Availability

The original contributions presented in the study are included in the article/Supplementary Material, further inquiries can be directed to the corresponding author.
